# The Phenomenology of Primary Orthostatic Tremor

**DOI:** 10.1002/mdc3.13454

**Published:** 2022-05-02

**Authors:** Bart E.K.S. Swinnen, Hanneke de Waal, Arthur W.G. Buijink, Rob M.A. de Bie, Anne‐Fleur van Rootselaar

**Affiliations:** ^1^ Department of Neurology Amsterdam University Medical Centers, Amsterdam Neuroscience, University of Amsterdam Amsterdam The Netherlands; ^2^ Department of Clinical Neurophysiology Amsterdam University Medical Centers, Amsterdam Neuroscience, University of Amsterdam Amsterdam The Netherlands

**Keywords:** orthostatic tremor, phenotyping, ataxia

## Abstract

**Background:**

The presence and prevalence of several neurological signs in patients with primary orthostatic tremor have not been systematically studied.

**Objectives:**

To assess the prevalence of clinical features of primary orthostatic tremor.

**Methods:**

Video‐based assessment by four raters of standardized neurological examination of 11 patients with primary orthostatic tremor.

**Results:**

On standing, bent knees (7/11), hem sign (6/10), and a broad base of support (6/11) were the three most prevalent signs. Examination of gait revealed abnormal tandem gait (9/11) and bent knees (6/11) as the most prevalent clinical signs. In the arms, none of the patients displayed bradykinesia, ataxia, or dystonia. In the legs, ataxia was absent in all patients and bradykinesia was present in only one patient.

**Conclusions:**

Abnormal tandem gait, bent knees, hem sign, and broad base on standing are the most prevalent clinical signs in primary orthostatic tremor. We did not encounter clear extrapyramidal or unequivocal cerebellar signs.

Primary orthostatic tremor (POT) is a rare idiopathic progressive neurological disorder characterized by a high frequency (>13 Hz) tremor in the leg muscles on standing.^1^, ^2^ Patients complain of instability on standing, with a normal to minimally affected gait.^3^, ^4^ Patients with POT are often severely disabled, there is no cure and treatment with medication or deep brain stimulation is often unsatisfactory.^3^

Several clinical signs have been established in POT. These include the helicopter sign, tremor of the knees, and fine‐amplitude rippling of leg muscles on standing.^3^, ^5^ A postural arm tremor with features reminiscent of essential tremor is present in the majority (50%–90%) of patients.^6^, ^7^, ^8^ Recently, the hem sign (ie, fast trembling of the hem of the skirt or long shirt covering the thigh on standing) has been reported in POT, but its prevalence is unknown.^9^ In the original descriptions of POT, abnormal tandem gait as well as a broad base of support and toe clawing on standing have been described.^10^, ^11^ However, presence and prevalence of these features have not been assessed systematically.

Whereas initial reports describe the absence of ataxia and bradykinesia in POT, several recent reports indicate the potential presence of cerebellar ataxia.^10^, ^11^, ^12^, ^13^, ^14^, ^15^ These mainly comprise of features of gait such as reduced step length and increased step width, but also a certain degree of appendicular ataxia has been suggested. These observations are contradicted by case series demonstrating only very rare occurrence of ataxia.^16^, ^17^, ^18^ Hence, presence of cerebellar ataxia in POT is still a matter of debate.^19^

We aimed to assess the prevalence of suggested clinical features of POT including the hem sign, abnormal tandem gait, broad base of support, and toe clawing, as well as the prevalence of extrapyramidal and cerebellar signs.

## Methods

At the annual meeting of the Dutch orthostatic tremor patient association on May 24th 2019 patients with POT were asked to undergo a video‐taped clinical examination. Eleven patients with POT consented and underwent a standardized neurological examination by a movement disorders expert (RMAB). Video recordings were independently assessed by two movement disorders experts (A.F.R. and B.E.K.S.S.) and two residents (A.W.G.B. and H.W.) scoring the presence or absence of an exhaustive list of predefined clinical signs. In case the rating by one assessor was different from the three others, the majority's rating was used. For features with equal ratings (ie, the sign being absent by two raters and present by the two others) a majority's rating was reached through a consensus meeting among the four raters. For each clinical sign the prevalence was determined by standard computations. To assess interrater reliability, for each clinical sign Light's Kappa and agreement were computed using RStudio (RStudio Team (2020). RStudio: Integrated Development for R. RStudio, PBC, Boston, MA URL http://www.rstudio.com/).

## Results

The patient characteristics are displayed in Table 1. Mean age at assessment was 68 years (range 55–78) with a mean disease duration of 20 years (range 7–24). Diagnosis of orthostatic tremor was confirmed by surface electromyography (EMG) tremor recording in all patients. At the moment of assessment most patients were taking medication for orthostatic tremor, of which perampanel was the most frequent.

**Table 1:** Patient characteristics

Patient characteristics (n = 11)	
Sex (M/F)	3/8
Age (mean and range), y^a^	68 (55–78)
Age at onset (mean and range), y	48 (25–65)
Disease duration (mean and range), y^a^	20 (7–34)
EMG confirmation	11^b^
Treatment^a^	
Perampanel	6
Clonazepam	4
Propranolol	2
Dual therapy	3^c^
None	2

^a^ At moment of evaluation.

^b^ In one patient only surface EMG with sound recording was performed.

^c^ Perampanel combined with clonazepam (two patients) or propranolol (one patient).

Abbreviation: EMG, electromyography.

Results are summarized in Table 2. On examination in the standing position, presence of bent knees (7/11), hem sign (6/10, Video 1) and a broad base of support (6/11, Video 1) were the most prevalent signs (Table 2). Of note, in one patient the hem sign could not be assessed as tight clothing was worn. Visible tremor was equally present in the legs (5/11) and arms (5/11), and less frequently in the trunk (4/11). Clawing of the toes during stance was noted in four patients (Video 1). Interrater reliability was moderate to substantial (ie, κ, 0.41–0.80) for all features, but slight to fair (ie, κ, 0.00–0.40) for broad base of support and toe clawing. Interrater reliability was highest for bent knees (0.75) and lowest for toe clawing (0.01).

**Table 2:** Standardized neurological examination of 11 primary orthostatic tremor patients by four raters

Condition	Prevalence – n	Interrater reliability		
		Light's κ		Agreement (%)
		κ	*P* value	
Standing				
Difficulty standing up	1	0.50	0.99	90.9
Tremor legs	5	0.59	0.57	63.6
Tremor arms	5	0.42	0.67	45.5
Tremor trunk	4	0.54	0.58	54.5
Broad base	6	0.21	0.79	27.3
Hem sign^a^ ^,^ ^b^	6	0.59	0.60	60.0
Bent knees	7	0.75	0.43	72.7
Toe clawing	4	0.01	0.99	9.1
Walking				
Difficulty starting or stopping	0	N/A	N/A	90.9
Hopping on start	0	N/A	N/A	72.9
Unstable	3	0.35	0.88	54.5
Decreased stride length	2	0.69	0.88	81.8
Broad base	2	0.18	0.97	54.5
Decreased arm swing	4	0.57	0.65	63.6
Clawed toes	3	N/A	N/A	54.5
Bent knees	6	0.44	0.62	45.5
Difficulty walking backward	3	0.54	0.89	72.7
Multi‐step turning	1	0.08	0.97	45.5
Loss of balance on turning	1	0.50	0.99	90.9
Side steps on tandem gait	9	1.00	0.80	100.0
Arms – Examination				
Outstretched; tremor^a^	3	0.83	0.73	90.0
Outstretched; dystonia^a^	0	N/A	N/A	100.0
Wing‐beating posture; tremor	4	0.90	0.50	90.9
Wing‐beating posture; dystonia	0	N/A	N/A	90.9
Finger tracking; dysmetria	4	0.21	0.90	36.4
Finger tracking; ataxia	0	N/A	N/A	63.6
Finger tracking; tremor	4	0.21	0.90	45.5
Finger tapping; bradykinesia	0	0.71	0.95	81.8
Pro‐supination; bradykinesia	0	N/A	N/A	90.0
Fists open‐closed; bradykinesia	0	N/A	N/A	90.9
Legs—Examination				
Heel‐to‐shin; dysmetria	0	N/A	N/A	100.0
Heel‐to‐shin; ataxia	0	N/A	N/A	90.0
Heel‐to‐shin; paretic	0	N/A	N/A	100.0
Foot tapping; bradykinesia	1	0.37	0.99	81.8
Leg agility; bradykinesia^a^	0	N/A	N/A	100.0

^a^ Assessable in 10 of 11 patients

^b^ According to the original description by Vidailhet et al.^9^ N/A, not applicable.

**Video 1:** Neurological signs in primary orthostatic tremor. Part A (00:00–00:23): broad base of support; two cases with broad and two cases with normal base of support. Part B (00:23–01:05): hem sign; two cases with and two cases without hem sign. Part C (01:05–01:29): toe clawing; two cases with and two cases without toe clawing (one of the latter being controversial). Part D (01:29–02:11): abnormal tandem gait; two cases with abnormal and two cases with normal tandem gait. Part E (02:11–02:45): dysmetria with finger tracking; two cases with dysmetria and two cases without dysmetria on finger tracking (one of each being controversial). For “controversial” signs a majority's rating was to be determined during the consensus meeting.

Examination of gait revealed abnormal tandem gait (9/11, Video 1), bent knees (6/11) and bilateral decreased arm swing (4/11) as the most prevalent clinical signs. Only a minority of patients exhibited an unstable gait (3/11), a broad base (2/11), and decreased stride length (2/11). Interrater reliability was excellent (ie, κ, 1.00) for tandem gait, whereas only slight (ie, κ, 0.00–0.20) for broad base and multi‐step turning.

None of the patients displayed bradykinesia, ataxia, or dystonia in the arms. Postural tremor was present in three (arms outstretched) to four (wing‐beating posture) patients with perfect (ie, κ, 0.81–1.00) interrater reliability. Kinetic tremor was present in four patients with fair (ie, κ, 0.21–0.40) interrater reliability. Postural and kinetic arm tremor, if present, had a small amplitude and medium to high frequency. Dysmetria during finger tracking was noted in four patients, but with only fair interrater reliability (Video 1).

When examining the legs, ataxia was absent in all patients and bradykinesia was present in only one patient.

## Discussion

POT is mostly affecting a patient's ability to stand still. Several clinical signs on standing have been postulated, but prevalence was largely unknown. This series indicates the hem sign and bent knees to be present in the majority (around 60%) of patients with good interrater reliability, making these confident features of POT. The hem sign is more prevalent than visible tremor in any particular body part (eg, visible tremor in the legs), probably because the hem sign constitutes the congregate of tremor in any body part. Of note, the hem sign can best be assessed when wearing loose clothes. Toe clawing was seen in around a third of patients and exhibits very low interrater agreement. This probably relates to the difficult discrimination, because of overlap between physiological variants of toe anatomy in this age group and pathological toe clawing in POT. Indicative features might be absence of toe clawing at rest and accompanying whitening of the toe tips indicating active downward pressure on the toes (see first case in Video 1, part C). As yet, toe clawing is not to be regarded as a sensitive or specific sign of POT and requires further investigation.

Apart from tremor, no clear extrapyramidal (ie, bradykinesia or dystonia) or cerebellar features (ie, ataxia) are present in the arms or legs. Dysmetria in the arms was noted in around a third of patients, however with low interrater agreement and is probably because of superimposed action tremor, which is also present in around a third of patients.

During walking certain additional features were present at low prevalence. Around a third of patients exhibited a decreased arm swing. Because there were no appendicular extrapyramidal signs and the reduced arm swing was bilateral, this is probably to be regarded as a compensatory phenomenon reflecting cautiousness of gait. Gait was less often broad‐based compared to stance, indicating a better stability while walking compared to standing.

The high prevalence of abnormal tandem gait and a broad base of support when standing raises the possibility of cerebellar ataxia underlying certain POT features as has been suggested by others. Additionally, some patients exhibited gait features that are also present in cerebellar ataxic gait (eg, instability, broad base, and decreased stride length). However, the observations in this case series are most compatible with cerebellar ataxia not constituting a phenomenological role in POT. On specific testing appendicular ataxia was absent in our patients, whereas the characteristic tremor in POT is most prominently present in the limbs. Additionally, except for abnormal tandem gait and broad base of support on standing, prevalence of cerebellar features is very low. Moreover, several “cerebellar” signs are not specific for cerebellar dysfunction because these may be present because of a variety of other reasons. In the case of POT, the instability induced by the rhythmic tremor probably induces a compensatory broadened base of support and shortened stride length (as one would do when walking on ice or a moving surface). The high prevalence of abnormal tandem gait in our case series probably merely reflects the surge of tremor severity when performing this task (ie, at low speed with a lot of support time). This lack of specificity of several appendicular and axial tests might explain the high prevalence of ataxic features in previous reports.^12^, ^13^, ^14^, ^15^ On the one hand, ataxia has been reported to be present in up to 50% of control patients indicating the low specificity for cerebellar dysfunction.^14^ On the other hand, on certain used ataxia rating scales contamination by tremor is very likely (eg, oscillating movement during finger to nose or abnormal tandem gait in the BARS).^14^ Moreover, in these reports POT patients scored very low on ataxia rating scales, indicating the limitedness of this observation.^14^ Although not assessed in our patients, gait performance in POT is known to improve with gait speed, especially with fast walking. This is not in line with cerebellar ataxia underlying the POT gait disturbance as cerebellar ataxia features become worse with fast walking.^20^ Nevertheless, a certain cerebellar component in the POT gait cannot be excluded entirely as high spatiotemporal gait variability has been shown previously.^15^ Moreover, the absence of clinical cerebellar ataxia does not preclude the cerebellum from contributing to the disease mechanism, as several studies have demonstrated functional and structural cerebellar alterations in POT and as several locations structurally connected to the cerebellum have been demonstrated to cause secondary OT.^21^, ^22^, ^23^ Nevertheless based on imaging studies, brain regions other than the cerebellum may be involved as well.^22^

Altogether, cerebellar ataxia seems not to be a clear nor consistent feature of POT. In fact, presence of clear ataxia is not compatible with a diagnosis of POT, because it indicates a secondary OT and should instigate diagnostic tests for secondary causes of OT.

There are some limitations to our study. No control individuals have been assessed, precluding determination of specificity of clinical features. Whereas interrater agreement was fair to good for most clinical signs, it was insufficient for some (ie, toe clawing, broad base when walking, multi‐step turning, and dysmetria on finger tracking). This indicates a limited usefulness in clinical practice when assessing these features. Although the sample size is considerably larger than most previous studies, it is still relatively small. Hence, features could not be related to patient characteristics including disease duration, disease severity, and effects of medication.

When considering all observations together, the hem sign, abnormal tandem gait, and bent knees and broad base on standing are among the most prevalent clinical features in POT. There are no clear extrapyramidal or cerebellar signs. POT seems to be a pure tremor disorder, in line with initial descriptions.^10^, ^11^

## Disclosures

**Ethical Compliance Statement:** The authors confirm that the approval of an institutional review board was not required for this work. Signed informed consents have been obtained from the patients. We confirm that we have read the Journal's position on issues involved in ethical publication and affirm that this work is consistent with those guidelines.

**Funding Sources and Conflicts of Interest:** The authors declare no funding or competing interests.

**Financial Disclosures for the Previous 12 Months:** The authors declare no competing interests.

## Author Roles

(1) Research project: A. Conception, B. Organization, C. Execution; (2) Statistical Analysis: A. Design, B. Execution, C. Review and Critique; (3) Manuscript: A. Writing of the First Draft, B. Review and Critique.

B.E.K.S.S.: 1C, 2A, 2B, 3A

H.W.: 1A, 1B, 1C, 2C, 2B

A.W.G.B.: 1C, 2C, 3B

R.M.A.B.: 1A, 1B, 1C, 2C, 3B

A.F.R.: 1A, 1B, 1C, 2C, 3B

## References

[1] Deuschl G , Bain P , Brin M . Consensus statement of the movement disorder society on tremor. Mov Disord 1998;13:2–23. 10.1002/mds.870131303 9827589

[2] Bhatia KP , Bain P , Bajaj N , et al. Consensus statement on the classification of tremors. From the task force on tremor of the International Parkinson and Movement Disorder Society. Mov Disord 2018;33(1):75–87. 29193359 10.1002/mds.27121 PMC6530552

[3] Benito‐León J , Domingo‐Santos Á . Orthostatic tremor: An update on a rare entity. Tremor Other Hyperkinet Mov (N Y). 2016;6:411. 27713855 10.7916/D81N81BT PMC5039949

[4] Hassan A , van Gerpen JA . Orthostatic tremor and orthostatic myoclonus: Weight‐bearing hyperkinetic disorders: A systematic review, new insights, and unresolved questions. Tremor Other Hyperkinet Mov (N Y) 2016;6:417. 28105385 10.7916/D84X584K PMC5233784

[5] DeOrchis VS , Geyer HL , Herskovitz S . Teaching video neuroimages: Orthostatic tremor: The helicopter sign. Neurology 2013;80(14):e161. 23547274 10.1212/WNL.0b013e31828ab301

[6] Gerschlager W , Münchau A , Katzenschlager R , et al. Natural history and syndromic associations of orthostatic tremor: A review of 41 patients. Mov Disord 2004;19(7):788–795. 15254936 10.1002/mds.20132

[7] Piboolnurak P , Yu QP , Pullman SL . Clinical and neurophysiologic spectrum of orthostatic tremor: Case series of 26 subjects. Mov Disord 2005;20(11):1455–1461. 16037915 10.1002/mds.20588

[8] Ganos C , Maugest L , Apartis E , et al. The long‐term outcome of orthostatic tremor. J Neurol Neurosurg Psychiatry 2016;87(2):167–172. 25770124 10.1136/jnnp-2014-309942

[9] Vidailhet M , Roze E , Maugest L , Gallea C . Lessons I have learned from my patients: Everyday life with primary orthostatic tremor. J Clin Mov Disord 2017;4(1):10–13. 28101372 10.1186/s40734-016-0048-5 PMC5234118

[10] Britton TC , Thompson PD , van der Kamp W , et al. Primary orthostatic tremor: Further observations in six cases. J Neurol 1992;239(4):209–217. 1597687 10.1007/BF00839142

[11] Heilman KM . Orthostatic tremor. Arch Neurol 1984;41(8):880–881. 6466163 10.1001/archneur.1984.04050190086020

[12] Feil K , Böttcher N , Guri F , Krafczyk S , Schöberl F , Zwergal A , Strupp M . Long‐term course of orthostatic tremor in serial posturographic measurement. Park Relat Disord. 2015;21(8):905–910. 10.1016/j.parkreldis.2015.05.021 26071126

[13] Opri E , Hu W , Jabarkheel Z , et al. Gait characterization for patients with orthostatic tremor. Park Relat Disord 2020;71(January):23–27. 10.1016/j.parkreldis.2020.01.007 31981995

[14] Thompson R , Bhatti DE , Hellman A , et al. Ataxia prevalence in primary orthostatic tremor. Tremor and Other Hyperkinetic Movements 2020;10(1):1–9. 33362948 10.5334/tohm.570 PMC7747757

[15] Möhwald K , Wuehr M , Schenkel F , Feil K , Strupp M , Schniepp R . The gait disorder in primary orthostatic tremor. J Neurol 2020;267(s1):285–291. 10.1007/s00415-020-10177-y PMC7718181 32915312

[16] Hassan A , Ahlskog EJ , Matsumoto JY , Milber JM , Bower JH , Wilkinson JR . Orthostatic tremor – clinical, electrophysiologic, and treatment findings in 184 patients. Neurology 2016;86:458–464. 26747880 10.1212/WNL.0000000000002328

[17] Yaltho TC , Ondo WG . Orthostatic tremor: A review of 45 cases. Park Relat Disord. 2014;20(7):723–725. 10.1016/j.parkreldis.2014.03.013 24736049

[18] Mestre TA , Lang AE , Ferreira JJ , et al. Associated movement disorders in orthostatic tremor. J Neurol Neurosurg Psychiatry 2012;83(7):725–729. 22577231 10.1136/jnnp-2012-302436

[19] Benito‐León J , Domingo‐Santos A . Orthostatic tremor: Clinical, electrophysiologic, and treatment findings in 184 patients. Neurology 2016;87(3):341. 27432180 10.1212/WNL.0000000000002907

[20] Schniepp R , Möhwald K , Wuehr M . Gait ataxia in humans: Vestibular and cerebellar control of dynamic stability. J Neurol 2017;264:87–92. 28397001 10.1007/s00415-017-8482-3

[21] Gallea C , Popa T , García‐Lorenzo D , et al. Orthostatic tremor: A cerebellar pathology? Brain 2016;139(8):2182–2197. 27329770 10.1093/brain/aww140 PMC4958903

[22] Benito‐León J , Romero JP , Louis ED , Sánchez‐Ferro A , Matarazzo M , Molina‐Arjona JA , Mato‐Abad V . Diffusion tensor imaging in orthostatic tremor: A tract‐based spatial statistics study. Ann Clin Transl Neurol 2019;6(11):2212–2222. 31588694 10.1002/acn3.50916 PMC6856595

[23] Benito‐León J , Rodríguez J , Ortí‐Pareja M , Ayuso‐Peralta L , Jiménez‐Jiménez FJ , Molina JA . Symptomatic orthostatic tremor in pontine lesions. Neurology 1997;49(5):1439–1441. 9371935 10.1212/wnl.49.5.1439

